# Associations between non-discrimination and training policies and physicians’ attitudes and knowledge about sexual and gender minority patients: a comparison of physicians from two hospitals

**DOI:** 10.1186/s12889-016-2927-y

**Published:** 2016-03-12

**Authors:** Jennifer M. Jabson, Jason W. Mitchell, Benjamin Doty

**Affiliations:** 1 Department of Public Health, University of Tennessee, 390 HPER, 1914 Andy Holt Ave, Knoxville, TN 37996 USA; 2 Department of Public Health Sciences, University of Miami Miller School of Medicine, Miami, FL USA; 3 Johns Hopkins Bloomberg School of Public Health, Baltimore, MD USA

**Keywords:** Non-discrimination policy, Physicians’ attitudes and knowledge, Sexual and gender minorities

## Abstract

**Background:**

Some physicians lack knowledge and awareness about health issues specific to sexual and gender minority (SGM) individuals. To help improve this, hospitals have implemented policies that mandate non-discrimination and training to promote sexual and gender minority health. There is limited evidence about how such policies relate to physicians’ knowledge, attitudes, and gender and sexual minority affirmative practices.

**Method:**

A random sample of 1000 physicians was recruited from a complete list of physicians affiliated with one of two university Hospitals located in Tennessee and 180 physicians completed the survey concerning attitudes and knowledge about SGM individuals. Physicians were affiliated with either Hospital A that had not implemented policies for non-discrimination and training, or Hospital B that did.

**Results:**

Physicians held different attitudes about SGM patients than non-patients. Physicians affiliated with Hospital A held more negative attitudes about SGM individuals who were non-patients than physicians affiliated with Hospital B. There were no differences between the two hospitals in physicians’ attitudes and knowledge about SGM patients.

**Conclusion:**

Policies that mandate non-discrimination and training as they currently exist may not improve physicians’ attitudes and knowledge about SGM individuals. Additional research is needed to understand how these policies and trainings relate to physicians’ SGM affirmative practices.

## Background

Sexual (gay, lesbian, bisexual) and gender (i.e., transgender) minority (SGM) individuals report significant dissatisfaction with healthcare, interactions with providers, and have more unmet needs than their heterosexual counterparts. According to McNair and colleagues [1], sexual minority women are 85 % more likely to leave a primary care setting with unmet needs and 50 % less likely to receive healthcare that was needed [2], including preventive screenings such as annual physical exams and pelvic and cervical screening [3] than heterosexual women. Sexual minority women also perceive that physicians do not spend enough time with them compared to heterosexual women [4]. Sexual minority men also report less satisfaction with healthcare than heterosexual men [4]. Using a representative sample of sexual minority men, Clift and Kirby found that more sexual minority men reported disrespect from their medical doctor than heterosexual men [4]; 15 % of sexual minority men also felt that they did not get enough time with their provider compared to 7 % of heterosexual men.

Physicians’ awareness, knowledge, and attitudes, about SGM patients and non-patients may contribute to these problems. As noted in two studies, 44–63 % of physicians reported being unaware of having sexual minority patients in their practice [5, 6]. Physicians have also reported a lack of knowledge about the health concerns and issues faced by sexual minority patients. Such concerns include, but are not limited to, risk behaviors such as alcohol, tobacco, and substance use, sexual health education, mental health including anxiety and depression, as well as overweight/obesity [6, 7]. Dahan and colleagues and Westerstahl and colleagues indicate that 63–92 % of physicians report no knowledge about these health concerns or the unique health issues faced by SGM patients and non-patients [5, 6]. Homophobia and heterosexism also shape the expectation that all physicians treat all patients the same and that discrimination and bias do not influence medical treatment. However, in their seminal report on anti-gay discrimination in medicine, Schatz and colleagues found that sexual minority individuals received substandard care and denial of care due to physician’s discrimination based on patient sexual orientation [8]. In their report 88 % of respondents reported witnessing a physician make disparaging remarks about sexual minority patients because of their sexual orientation [8]. A more recent report by Grant and colleagues found that 28 % of patients who identified as transgender experienced verbal harassment in a medical care setting and 50 % reported providers lacked the knowledge needed to provide necessary medical care [9].

Physicians’ attitudes and knowledge about SGM individuals may vary by gender. In multiple studies published across several decades, females tend to show more positive attitudes about SGM than males [7, 10–12]. Using a national probability sample of heterosexual adults in the United States, Norton and colleagues (2014) found that females had significantly more positive attitudes about gender minority people as compared to their male counterparts. Larsen and colleagues (1980) reported similar findings where females held more positive attitudes about toward homosexuality than did males. One study conducted with medical students has shown that female medical students hold more favorable attitudes about sexual minority people than male medical students [7]. The previous studies did not involve medical physicians and it is not clear from these studies if gender differences would persist in a sample of physicians.

Legal rights have expanded to sexual and gender minorities in recent years. However, discrimination of SGM individuals remains present in healthcare settings, which may contribute to health disparities. One possible solution is policies at the level of the healthcare organization that make mandatory non-discrimination and training in SGM health [13]. Non-discrimination policy exists at the federal level; the Joint Commission for the Accreditation of Healthcare Organizations mandated the development and implementation of nondiscrimination policies in medical care and equal visitation rights for sexual and gender minorities [14]. Additionally, the Centers for Medicare and Medicaid Services has changed their Conditions of Participation requiring hospitals to allow “equal visitation for patients”, including SGMs [15]. However, whether or not mandates influence physician attitudes and knowledge is unknown. Further, training for physicians in SGM health issues has not been federally mandated.

The Human Rights Campaign developed the Healthcare Equality Index (HEI) to document and promote healthcare organizations’ voluntary policy implementation that mandates non-discrimination and training according to four core criteria [16]. The four core criteria are: 1. Patient non-discrimination policy written into the patient bill of rights that specifies non-discrimination to sexual and gender minorities and is communicated to patients in two formats; 2. Equal visitation for SGM patients and their non-biological family members; 3. Employment non-discrimination that specifies non-discrimination against SGM employees; and 4. Training in SGM patient centered care for physicians, non-physician healthcare providers, and staff. If a healthcare organization meets all four of the core criteria they earn the commendation of ‘criterion leader’ in SGM patient-care. If an organization does not meet any of the four core criteria, HEI provides the organization with recommendations to resolve unmet criteria.

The intention of the HEI is to recognize and promote SGM patient-centered care and safe healthcare settings in which SGM individuals can receive care. However, there is a dearth of published empirical evidence that informs the relationship between receipt of commendation given by the HEI and physicians’ attitudes and knowledge about SGM patients. In fact, to our knowledge there is no documentation of these relationships. The goal of the current study is to begin to address this gap. We hypothesize that physicians who were affiliated with a medical hospital that received HEI commendation as a criterion leader in SGM patient-centered care would have more positive attitudes, greater knowledge about SGM health issues, and better SGM affirmative practice than physicians affiliated with a hospital that did not receive such commendation.

## Methods

### Procedures

HEI ratings are made annually and for this study, ratings from 2013 were used to identify hospitals from which to recruit physicians. This was a cross-sectional study involving two hospitals. Hospital A is a 581-bed private, academic, Level I trauma hospital located in an urban area of East Tennessee (2013 population = 450,000). Hospital B is an 834-bed private, academic, Level I trauma hospital located in an urban area of middle Tennessee (2013 population = 658,602) [17, 18]. Both hospitals have received high rankings for performance in U.S. News and World Reports [19]. According to the 2014 Urban–rural Classification scheme [20], Hospital B is located in a medium metro metropolitan area compared to Hospital A that is located in a small metro metropolitan area.

To achieve a 31-57 % response rate, 1000 physicians were recruited via random sampling to obtain a sample size between 310 and 570 physicians. This sample size corresponded to greater than or equal to 80 % power in each dependent variable. A random sample of 1000 physicians was drawn from a complete list of physicians affiliated with one of two university Hospitals located in Tennessee; 500 from Hospital A (without commendation, meeting none of the four HEI criterion for non-discrimination policies and training) and 500 from Hospital B (with commendation for non-discrimination policies and training; achievement of all four HEI criterion). The names of all physicians affiliated with either of the two hospitals were entered into an excel spreadsheet. Then the excel function Rand() was used to assign a random number to all physicians in the list. The random numbers were then sorted numerically from lowest to highest and the first 500 physicians from each hospital were recruited to participate in the study. The 1000 randomly identified physicians were recruited to participate in this study via personalized invitation package delivered by mail. In an effort to guard against low response rate each invitation package included a personalized letter printed on university letterhead, addressed to the physician by name, and described the study and the study’s purpose to better understand physicians’ attitudes and beliefs about SGM patients. The personalized package also included a description of the compensation (i.e., random drawing for $200), a hard-copy version of the respondent-friendly survey, and the link to an online survey option [21]. The invitation encouraged participants to complete the survey either online or in hard copy and return using the enclosed self-addressed stamped envelope. This study was approved by the University of Tennessee Knoxville Institutional Review Board (protocol # 9395B).

### Measures

#### General attitudes toward lesbian, Gay, bisexual, and transgender Non-patients

Attitudes toward lesbians, gay men, bisexuals, (sexual minority) and transgender (gender minority) individuals (ATLG) were assessed with four separate measures. The ATLG and its individual subscales have shown consistent reliability. Cronbach’s alpha is .90 for the full scale and subscales were as follows: lesbian α = .84 and gay α = .83. In the current study, for the full scale, Cronbach’s alpha was .97. Cronbach’s alpha for each subscale in our data was as follows: lesbian α = .78, gay α = .76, bisexual α = .78, and transgender α = .70. The ATLG has been consistently correlated with theoretical constructs, with higher scores correlating with more negative attitudes, such as adherence to traditional beliefs, lack of contact with sexual minority individuals, and dogmatism [22]. Herek’s 6-item scale for “Attitudes toward Lesbians and Gays” was used and expanded to include bisexual and transgender individuals [22]. Individual items were set to a 5-point Likert scale (5 = strongly agree). Individual items included “Sex between two men is just plain wrong”, “I think male homosexuals (gays) are disgusting”, and “Male homosexuality is a natural expression of sexuality in men” [22]. The language for each item was modified according to each of the three sexual orientation sub-groups and gender minorities. Positive items were reverse-scored and items were summed such that higher scores indicated having more negative attitudes toward SGM individuals.

#### Physicians’ attitudes toward lesbian, Gay, bisexual, and transgender patients

Physicians’ attitudes about treating lesbian, gay, bisexual, (sexual minority) and transgender (gender minority) (LGBT) patients (ATLGBTP) was measured with a modified version of Harris and colleagues’ 6-item Questionnaire for Health Care Professionals [23, 24]. Individual statements included: “I would prefer not to provide care for LGBT patients”, “I would refuse care for a LGBT patient if I were aware that they identified as LGBT,” “I feel competent to provide care for LGBT patients”, “LGBT patients do not have any specific health needs,” “I feel I would be able to talk to a patient who identifies as LGBT in a sensitive and appropriate manner,” and “I believe my medical training adequately addressed the health needs of the LGBT population” [23, 24]. The ATLGBTP has shown modest reliability of .54 on Cronbach’s alpha. In the current study Cronbach’s alpha was .50. The number of scale items may have influenced its reliability. Results from the ATLGTP should be interpreted carefully; the minor modifications may have influenced the .04 difference in reliability between ours and the Cronbach’s alpha published by others [23]. Each of the 6 items was set to a 5-point Likert scale (5 = strongly agree). Positive items were reverse scored and all items were summed such that higher scores indicated more negative attitudes about treating LGBT patients.

#### Knowledge of lesbian, Gay, bisexual, and transgender patients

This measure includes 13 true/false items modified from Strong and colleagues’ (unpublished thesis) and Harris and colleagues’ questionnaire [24]. The KLGBT scale a Cronbach’s alpha level of .74 among health care professionals. The scale’s construct validity was determined by having different types of health professionals (e.g., psychologists, social workers) take the test. Scores on the test were significantly higher for health professionals than for people with less education. The KLGBT has correlated with theoretical constructs, with higher scores being associated with significantly less prejudice. Correlations on various measures have ranged from -.41 to -.61. [25] In this sample, KR-20 was used as the reliability estimate (appropriate for dichotomous items); KR-20 = .59. Individual items included but were not limited to: “Sex and gender have the same meaning”, “most homosexuals want to be members of the opposite sex”, “Lesbian, gay, bisexual, and transgender patients do not seek medical treatment as early as heterosexuals because of fear of discrimination”, and “most health care providers automatically make the assumption that their patient is heterosexual if they have not specifically addressed sexual orientation”. Correct responses were scored a 1 and incorrect responses were scored a 0; total responses were then summed. High scores indicated more knowledge about SGM individuals.

#### Gender and sexual minority affirmative practice scale

A modified version of the Gay Affirmative Practice (GAP) Scale, renamed for this study more inclusive (Gender and Sexual Minority Affirmative Practice Scale), was used to assess physicians’ attitudes and beliefs about the treatment of SGM patients and physicians’ behaviors in clinical setting with patients [26]. For this study this measure included 10 items set to a 5-point Likert scale (5 = strongly agree). The GAP scale has an overall Cronbach’s alpha level of .95 for the full scale. The two subscales (beliefs and behaviors) have demonstrated Cronbach’s alpha of .93 and .94, respectively, demonstrating high levels of reliability. Tests of the GAP’s construct validity have shown that each item loads at a minimum of .60 in the intended domain [26]. Individual items included but were not limited to the following statements: “In their practice with lesbian, gay, bisexual and transgender clients, practitioners should support the diverse make up of their families”, “Practitioners should verbalize respect for the lifestyles of lesbian, gay, bisexual and transgender clients” and “Practitioners should be knowledgeable about issues unique to lesbian, gay, bisexual and transgender individuals”. Five statements present in the original GAP were removed from the current study because they did not apply to physicians.

#### Demographic and medical practice characteristics

Physicians also answered questions regarding their age, gender, sexual orientation, and marital status. Physicians’ medical practice-related characteristics were documented with the following questions: “Please define your specialization” (open ended response), “How many years have you been practicing medicine?” (open-ended response), “What is your role at the hospital where you are employed?” (open-ended response), “How often do you provide direct care to patients?” (daily/weekly/less than monthly/monthly) and “Have you ever received focused training regarding lesbian, gay, bisexual and transgender patient care?” (yes/no).

### Analyses

Descriptive and summary statistics were calculated to describe the sample and dependent variables. To test for mean differences between each hospital, non-normally distributed dependent variables were analyzed using the Mann–Whitney U Test whereas the t-test was used for normally distributed dependent variables. Independent samples t-tests and Mann–Whitney U tests, respectively, were calculated for each independent variable to determine differences by hospitals in physicians’ attitudes, beliefs, and knowledge about SGM patients. Multivariate linear regression was used to test for associations between the Gender and sexual minority affirmative practice Scale and physicians’ general attitudes about non-patient SGM individuals and attitudes about SGM patients, with adjustment for demographic and medical specialization. The frequency and percent missing data for each dependent variable was calculated. Associations between missingness and physicians demographic characteristics were tested. Analyses were calculated using IBM SPSS Statistics for Mac, v22.

## Results

### Participant characteristics

Of the 1000 physicians recruited, 180 returned completed surveys (108 from Hospital A; 72 from Hospital B). The survey response rate was 18 %, significantly lower than the 31–57 % response rate reported by others who use similar strategies to recruit physician participation in survey research [21, 27, 28]. There was less than 10 % missing in all dependent variables (range: 1–6 %) and there were no significant associations between missingness and physicians demographic characteristics. Our study was underpowered due to small sample size (power = .43).

Table 1 summarizes the demographic characteristics of the full sample (*N* = 180) and by physicians’ location (Hospital A (*n* = 108), B (*n* = 72)). In the full sample, 171 (95 %) of physicians reported that they were aware, and 9 (5 %) were unaware, that patients in their practice identified as gay, lesbian, or bisexual, and 171 (95 %) reported that they were aware, and 9 (5 %) were unaware, that patients in their practice identified as transgender. One-hundred percent of the physicians affiliated with Hospital B reported awareness of SGM patients in their practices. The full sample was largely characterized as middle aged (M age = 50.1, SD = 11.2), male (66 %, *n* = 119), heterosexual (97 %, *n* = 174), and either married or partnered (90 %, *n* = 162). There was a 10 % difference in physicians’ gender between hospitals (Hospital A: 70 %; Hospital B: 60 %). The sample included physicians from 27 medical specialties, as categorized by the American Medical Association [29]. The most common specialties represented were: Internal Medicine (16 %), Surgery (11 %), Pediatrics (10 %), Obstetrics/Gynecology (7 %), Oncology (7 %), Family Medicine (7 %), and Cardiology (6 %). Other specialties, each representing less than 5 % of physicians in the sample, included: Allergy/Immunology, Anesthesiology, Dentistry, Dermatology, Emergency Medicine, Endocrinology, Geriatrics, Medical Genetics, Neurological Surgery, Neurology, Ophthalmology, Orthopedics, Otolaryngology, Pathology, Physical Medicine/Rehabilitation, Preventive Medicine, Psychiatry, Radiology, Urology, and Other. Physicians reported 20.5 average years of experience (SD = 11.9); the sub-sample from Hospital A was slightly more experienced than the sub-sample from Hospital B. The majority of physicians in this sample provided direct care to patients daily. In the full sample, 83 % (*n* =149) of physicians provided direct care daily, compared to 89 % (*n* = 96) at Hospital A and 74 % (*n* = 53) at Hospital B. Less than 20 % of physicians at both Hospitals (A: *n* = 17; B: *n* = 10) reported having had training in lesbian, gay, bisexual, and/or transgender patient care. The location and time of this training was not indicated.

**Table 1:** Physicians’ demographic characteristics by hospital

	Combined (*N* = 180)		Hospital A without policy/training (*N* = 108)		Hospital B with policy/training (*N* = 72)	
	*N*, %		*n*, %			
Age (M ± SD)	50.1 ± 11.2		51.2 + 10.9		48.5 + 11.3	
Yrs Experience (M ± SD)	20.5 ± 11.9		21.1 ± 12.0		19.5 ± 11.8	
Sex						
Male	119	66.1	76	70.4	43	59.7
Female	60	33.3	31	28.7	29	40.3
Transgender	0	0.0	0	0.0	0	0.0
Declined Response	1	0.6	1	0.9	0	0.0
Orientation						
Straight	174	96.7	103	95.4	71	98.6
Gay	4	2.2	3	2.8	1	1.4
Bisexual	0	0.0	0	0.0	0	0.0
Declined Response	1	0.6	4	0.9	0	0.0
Marital Status						
Married	161	89.4	97	89.8	64	88.9
Partnered	1	0.6	0	0.0	1	1.4
Single	9	5.0	6	5.6	3	4.2
Divorced	7	3.9	5	4.6	2	2.8
Widowed	1	0.6	0	0.0	1	1.4
Direct Care						
Provision						
Daily	149	82.8	96	88.9	53	73.6
Weekly	23	12.8	7	6.5	16	22.2
Monthly	2	1.1	1	0.9	1	1.4
Less than Monthly	4	2.2	4	3.7	0	0.0
Aware of gay, lesbian and bisexual						
patients in practice	171	95.0	99	91.7	72	100.0
Unaware	9	5.0	9	8.3	0	0.0
Aware of transgender patients in practice	171	95.0	99	91.7	72	100.0
Unaware	9	5.0	9	8.3	0	0.0
Received Training in LGBT Patient Care (Yes)	27	15.0	17	15.7	10	13.9

### Physicians’ beliefs

#### General attitudes toward lesbian, Gay, bisexual, and transgender non-patients

The descriptive statistics for each measure of physicians’ attitudes and knowledge are presented in Table 2. In the full sample of physicians, ATLGBT scores ranged from 12 to 55 with a mean score of 26.9 (SD = 9.9). Overall, physicians held the greatest negative attitudes toward bisexual non-patients (*M* = 7.0, SD = 2.7) and the least negative attitudes toward transgender non-patients (*M* = 6.5, SD = 2.4). Physicians’ attitudes were identical toward gay non-patients (*M* = 6.7, SD = 2.6) and lesbian non-patients (*M* = 6.7, SD = 2.6).

**Table 2:** Descriptive statistics for physicians’ attitudes toward sexual and gender minority individuals

	Full Sample				Hospital A without policy/training				Hospital B with policy/training				
	*N*	Mean/Median	SD	Range	*N*	Mean/Median	SD	Range	*N*	Mean/Median	SD	Range	Mann–Whitney Tests/t-tests
Attitudes Toward Lesbians and Gay Men Non-Patients	176	26.9	9.9	12–55	105	28.8	2.6	12--55	71	24.1	9.0	12–49	*z* = −3.04, *p* = .002
Attitudes Toward Gay Men Subscale	179	6.7	2.6	3–15	108	7.2	2.6	3–15	71	6.0	2.4	3–13	*z* = −3.16, *p* = .002
Attitudes Toward Lesbians Subscale	179	6.7	2.6	3–15	107	7.2	2.6	3–15	72	6.0	2.5	3–13	*z* = −3.10, *p* = .002
Attitudes Toward Bisexuals Subscale	178	7.0	2.7	3–14	106	7.5	2.7	3–14	72	6.3	2.6	3–14	*z* = −3.0, *p* = .003
Attitudes Toward Transgender People Subscale	178	6.5	2.4	3–12	106	6.9	2.4	3–12	72	6.0	2.2	3–11	*z* = −2.5, *p* = .012
Attitudes Toward Lesbian, Gay, Bisexual, and Transgender Patients	176	12.2	2.9	6–21	106	12.0	2.8	6–20	70	12.4	3.2	6–21	t(174) = −0.92, *p* = .357
Sexual and Gender Minority Affirmative Practice Scale	173	42.1	8.5	11–55	104	42.0	8.6	11–55	69	42.3	8.3	20–55	*z* = −0.44, *p* = .661
Knowledge of Lesbian, Gay, Bisexual and Transgender Non-Patients	169	12.1	1.3	6–13	102	12.0	1.3	6–13	67	12.2	1.5	7–13	*z* = −1.68, *p* = .092

Physicians’ attitudes toward SGM non-patients were statistically different by Hospital; Hospital A had more negative attitudes (Md. = 30, *n* = 105) than Hospital B (Md. = 23, *n* = 71) (U = 2720.5, *z* = −3.04, *p* = .002). Physicians’ attitudes also varied by sub-scale. Physicians at Hospital A had more negative attitudes toward SGM non-patients than physicians at Hospital B (Table 2). Physicians’ attitudes about transgender non-patients were more negative at Hospital A than B (A: Md. = 7, *n* = 106; B: Md. = 6, *n* = 72; U = 2971.5, *z* = −2.52, *p* = .012).

#### Attitudes toward lesbian, Gay, bisexual, and transgender patients

Overall, the mean score for ATLGBTP was 12.2 (SD 2.9). Physicians’ attitudes about SGM patients did not vary by hospital (t (174) = −0.92, *p* = .357).

#### Knowledge of lesbian, Gay, bisexual, and transgender patients

Average score of KLGBT was 12.1 (SD 1.3). Physicians’ knowledge about LGBT patients did not vary by hospital (*U* = 2938.5, *z* = −1.68, *p* = .092).

#### Gender and sexual minority affirmative practice

The average GAP score was 42.1 (SD 8.5). There was no difference in physicians’ gender and sexual minority affirmative practice by hospital (*U* = 3447.0, *z* = −.044, *p* = .661).

## Discussion

In 2011, the Institute of Medicine published “The Health of Lesbian, Gay, Bisexual, and Transgender People: Building a foundation for better understanding” to raise awareness with a call for action to researchers to address the specific health and health care needs of SGM people [30]. The goal of the present study was to investigate how fulfilling the four core criterion and receiving HEI commendation, including policies that mandate non-discrimination policy and training for physicians, relates to physicians’ attitudes and knowledge about SGM patients.

Our findings provided partial support for our hypotheses. Physicians’ attitudes about SGM non-patients were less negative at Hospital A, the hospital with HEI commendation and non-discrimination policy and training. However, no differences were found in physicians’ attitudes and knowledge about SGM patients or gender and sexual minority affirmative practice between physicians at either of the two hospitals. Most physicians (95 %) in the sample were aware of SGM patients in their practices. This proportion is significantly higher than the findings reported by Westerstahl and colleagues and Dahan and colleagues who reported that 35–56 % of physicians, respectively, were aware of sexual minority patients in their practices [5, 6]. Elevated awareness among the physicians in our study could reflect a larger social change toward greater awareness. This type of large-scale change could be a positive first step toward improving quality of care received by SGM patients; physician’s awareness of SGM patients is a first step in addressing the unique medical care and treatment needs experienced by SGM patients.

The physicians who participated in the current study held less negative attitudes about sexual minority non-patients and sexual minority patients, than those reported by others. Compared to a randomly selected sample of non-physicians [31], the physicians in the current study reported less negative attitudes about non-patients who identified as gay (*M* = 8.6 vs *M* = 6.7) or lesbian (*M* = 8.5 vs *M* = 6.7). Compared to a sample of social workers, psychologists, and nurses the physicians in the current study also held significantly less negative attitudes about patients who identified as a sexual minority (*M* = 49.4 vs *M* = 12.2) [24]. Comparisons of attitudes about patients who identify as transgender were not possible. These differences in attitudes could be attributed to the difference in samples; neither Herek [31] nor Harris et al. [24] samples included medical physicians. It is possible that there is something about physician-focused medical education that could result in more positive attitudes about sexual minority individuals. However, it may be more likely the case that nationally attitudes about sexual minority individuals have been steadily improving nationally [32]. In a study of physician’s attitudes about sexual minorities, Smith and Mathews demonstrated a 39 % decrease in physician’s negative attitudes and stigma about sexual minority individuals from 1982 to 2007 [33]. It is possible that the less negative attitudes reported by physicians in the current sample could be an artifact of the growing, wide-scale, and positive changes in attitudes about sexual minority individuals.

We found some evidence of gender differences. The proportion of females at Hospital B (40 %) was greater than Hospital A (29 %), and it is possible that gender contributed to the differences in attitudes about SGM individuals (non-patients) between the two hospitals. Interestingly, the possibility of a gender effect was not evident in any other measures.

Differences in physicians time in providing direct care between Hospital A and Hospital B were noted, which also aligned with their attitudes toward SGM non-patients. Unlike Hospital A, Hospital B is a research hospital. It is possible that physicians at Hospital B had less time to provide direct care because of their involvement in research activities, which may have included direct interaction with study participants outside of the hospital setting (e.g., in the community, schools, online). Their involvement in these types of research activities could also help explain why they had more positive attitudes toward SGM non-patients.

Physicians’ attitudes about treating gender and sexual minority patients did not differ by hospital in this study. This similarity may be an artifact of social desirability; physicians have repeatedly self-reported that they treat all patients equally [34, 35]. The measure used in the current study asked physicians questions about preference to care for, refusal of care for, and inability to talk with, gender and sexual minority patients. To better understand these attitudes, and their existence in HEI criterion leader designated and undesignated facilities, a study design that removes the threat of social desirability must be used. One such strategy involves direct or recorded observations of physicians’ interaction with gender and sexual minority patients. This approach could help determine if differences exist in physicians’ attitudes toward gender and sexual minority patients by observing their interactions with patients.

The geopolitical context of a region in which physicians reside and practice could also have influenced the findings [24, 31]. However, the current study was conducted in the state of Tennessee; a state where many state level policies to protect SGM individuals’ access to healthcare, health and wellbeing do not exist. For instance, at the time that this study was conducted Tennessee did not support marriage equality, non-discrimination in housing based on sexual orientation or gender identity, second-parent or step-parent adoption for same-sex couples, employment discrimination based on sexual orientation or gender identity, discrimination in schools or anti-bullying, transgender healthcare or gender marker change. There is no evidence available at this time to support that state level policy or other geopolitical characteristics of the region positively biased physician’s attitudes and knowledge to make them less negative.

Another possibility to help explain these findings stems from the type and frequency of training that physicians received at their hospital. Less than 20 % of physicians at either Hospital in this study reported having had training in SGM patient care. Although Hospital B had earned the Health Equality Index (HEI) commendation of ‘criterion leader’ in sexual and gender minority patient-care centeredness, it appears that not all physicians from this hospital had received this specific training. This calls into question how often, and at what intervals, physicians receive training on implementing SGM patient-care centeredness, particularly among those who newly join a hospital and how often hospitals (and other healthcare centers) should provide such trainings to their physicians, staff and other healthcare providers. In addition, some physicians may have received training in SGM patient care and non-discriminatory practices before joining their current hospital; this may in part explain why a higher proportion of physicians at Hospital A (non-commendation) indicated they received training compared to those at Hospital B (commendation).

The lack of difference in physicians’ attitudes and knowledge by hospital could represent lack of awareness about and purpose of the HEI criterion leader designation. Advocates for the HEI could argue that physician lack of awareness is addressed with the fourth core criterion which states that physicians (and other providers and staff) receive training in SGM patient-centered care. However our results suggest that the fourth core criterion may not have the impact intended as only 15 % of physicians in this study recall receiving this training.

### Limitations

Despite the use of evidence-based approaches to guard against low response rate [21], the low response rate for this study is a limitation; there are several possible reasons for the low rate. First, the survey was included in a package of materials that described the study and requested consent from respondents. In this package of materials the purpose of the study, to understand physician’s attitudes and beliefs about SGM patients, was clearly stated. It is possible that the low response rate reflects physicians disregard for and lack of awareness of SGM patient care issues. Second, Hospital A was located in the same region as the study’s principal investigator and is affiliated with the area’s most prominent academic institution. This may have motivated physicians affiliated with Hospital A to participate in the study at a greater rate than those affiliated with Hospital B. Future efforts to involve physicians in survey research should consider the use of unconditional incentives to improve response rates. Abdulaziz and colleagues recently showed that in a national survey of physicians, using unconditional incentives boosted participation to more than 60 % [36]. The use of a cross-sectional study design with a convenience sample is also a limitation because it does not allow for casual inference or generalizability of these findings to all physicians in Hospitals A or B, the state of Tennessee or elsewhere. Further, social desirability may have influenced the sample and the findings. It is also possible that physicians with an interest in SGM health or who held SGM in a positive regard were more likely to respond to the survey than physicians who did not share these attitudes. According to the 2013 HEI, Hospital B, the HEI criterion leader in SGM patient-centered care, has policy that includes all four HEI criteria, including training for physicians, other care providers, and staff [16]. According to electronic materials publicly available online, Hospital B’s patient bill of rights specifies non-discrimination against SGM patients, however Hospital A’s patient bill of rights does not. The absence of non-discrimination language at Hospital A may contribute to the policy-practice gap. Further, in-depth analysis of policy materials at each facility could have allowed for deeper consideration for how policy relates to physicians attitudes and knowledge. Regretfully our study is limited because the necessary documents for such an analysis were unavailable. Additionally, our study was limited by the lack of in-depth analysis of the training materials used to fulfill the fourth core criterion. A range of trainings are available to physicians in gender and sexual minority patient-centered care, including online trainings and classroom trainings of varying lengths and intensity. Training differences with respect to content, duration, and delivery modality could influence the effect on physician attitudes and knowledge. Future research should address these limitations by collecting data from physicians anonymously, using a larger, nationally representative sample and by conducting in-depth analyses of policies and trainings in gender and sexual minority patient-centered care.

The HEI commendation for achieving the four core criteria is intended to improve health care for, and guide SGM patients to, high quality, safe sources of health care. Unfortunately, our findings do not give us faith that having an HEI commendation is indicative of more positive attitudes toward SGM patients, more SGM affirmative practice, or more knowledge of SGM patients. This is concerning because many national organizations promote HEI designated healthcare organizations to SGM patients and these patients rely on HEI commendations to make informed choices about where to receive safe health care. Despite our findings, we do believe that institutional anti-discrimination policy and policy that mandates trainings about SGM healthcare are important for physicians. We recommend that all healthcare facilities provide and require their physicians, staff and other healthcare providers to be trained on how best to provide SGM patient-care centeredness. Within these healthcare facilities, we also recommend that SGM patient outcomes along with their medical providers be rigorously evaluated to monitor whether the providers training translates to mastering these skills in order for SGM patients to receive the most appropriate care for their respective needs.

## Conclusion

The HEI aims to reduce discrimination and improve the healthcare milieu for the purpose of reducing health disparities experienced by gender and sexual minorities and improving quality of their healthcare. Our findings provide a first step, of many required, toward understanding possible associations between the criterion leader designation and physician attitudes and knowledge. Unfortunately, our results suggest that, at least in the case of the HEI criterion leader facility included in this study, fulfilling the four core criteria and earning the criterion leader designation may not produce the changes intended by the HEI and criterion leader designation. This is worrisome because the HEI criterion leader designation is used to promote ‘safe’, gender and sexual minority patient-centered healthcare environments to gender and sexual minority patients. However, if the education mandated by the fourth core criteria does not influence physicians’ attitudes and knowledge, gender and sexual minority patients could be directed to healthcare environments that may not have any more knowledge or better attitudes than healthcare settings without the HEI criterion leader designation. Rigorous evaluations need to be conducted across multiple facilities to further assess the impact on healthcare facilities of fulfilling the HEI four core criteria.

Physicians are not islands and they practice in social and political contexts that shape their experiences. Consequently it is inadequate for future work to focus exclusively on physicians’ attitudes and knowledge while ignoring the complex multi-level factors that influence medical practice and healthcare for SGM patients. Future work in this area should involve innovative, rigorous, multi-level assessment of patient-, provider-, system-, and policy-level factors that influence implementation of SGM affirming healthcare practice. For instance, Bronfenbrenner posits a multi-level ecological model where individual, dyadic, and community and policy contribute to physician’s knowledge, attitudes, and willingness to practice culturally appropriate care to sexual minority and gender minority patients [37]. And while there is a paucity of empirical evidence that describes the synergistic, accumulative, effect of the hierarchical levels, this could be a strong theoretical approach to use for future studies in this area. Another possible strategy recommended by McNair and colleagues involves the development, widespread implementation, and subsequent evaluation of informative, evidence-based guidelines for primary care with SGM patients [1]. Establishing guidelines and accompanying policy that mandates their use could significantly improve SGM affirming healthcare and could ultimately improve outcomes and reduce disparities in health.
